# Intravascular ultrasound characteristics in patients with intermediate coronary lesions and borderline fractional flow reserve measurements

**DOI:** 10.1097/MD.0000000000011901

**Published:** 2018-08-24

**Authors:** Hyoung-Mo Yang, Hong-Seok Lim, Kyoung-Woo Seo, Byoung-Joo Choi, So-Yeon Choi, Myeong-Ho Yoon, Gyo-Seung Hwang, Seung-Jea Tahk

**Affiliations:** Department of Cardiology, Ajou University School of Medicine, Suwon, Republic of Korea.

**Keywords:** borderline fractional flow reserve, intravascular ultrasound, plaque volume

## Abstract

Revascularization of borderline fractional flow reserve (FFR) is controversial and the morphologic characteristics of borderline FFR lesions are not well known. The objective of this study was to determine the intravascular ultrasound (IVUS) characteristics in intermediate coronary lesions with borderline FFR in patients with intermediate coronary artery stenosis (40%–70% diameter stenosis).

Both IVUS and FFR were performed in a total of 228 left anterior descending arteries. We divided them into 3 groups by FFR value: ischemic (n = 46, FFR < 0.75), borderline (n = 71, FFR 0.75 to ≤0.80), and non-ischemic (n = 111, FFR > 0.80). We compared the IVUS parameters, including minimum lumen area, lesion length, plaque burden, and volumetric analysis among the 3 groups.

In the IVUS analysis, the minimum lumen area was smaller (2.5 ± 0.6 vs. 2.7 ± 0.7 vs. 3.4 ± 1.2 mm^2^, *P* < .001); lesion length was longer (23.6 ± 8.4 vs. 23.6 ± 7.4 vs. 17.4 ± 6.8 mm, *P* < .001); plaque burden was larger (76.1 ± 9.6 vs. 73.9 ± 7.5 vs. 69.8 ± 9.5%, *P* < .001); plaque volume was larger (173.0 ± 78.3 vs. 167.7 ± 75.0 vs. 129.5 ± 79.1 mm^3^, *P* < .01); and percent atheroma volume was larger (57.9 ± 7.5 vs. 57.6 ± 6.6 vs. 53.9 ± 8.0%, *P* < .01) in the ischemic and borderline groups compared with the non-ischemic group, respectively. However, post-hoc analyses showed there were no significant differences between the ischemic and borderline group for all IVUS parameters.

There were no differences in IVUS characteristics between borderline and functionally significant FFR, but the amount of atheromatous plaque was more severe in these 2 groups than in the non-ischemic group.

## 1 Introduction

Fractional flow reserve (FFR) is an invasive method to assess myocardial ischemia, and FFR-guided treatment of coronary artery disease has shown good clinical outcomes.^[1–3]^ FFR has been validated with several noninvasive functional studies. It is well known that a lesion with FFR <0.75 is associated with myocardial ischemia, and FFR >0.80 indicates absence of inducible ischemia in the majority of patients.^[4–7]^ However, data indicate that it is debatable whether revascularization is needed in borderline FFR, which is defined as FFR 0.75 to 0.80.^[8–10]^ For the treatment of borderline FFR, the DEFER study deferred patients with borderline FFR using a cutoff value of 0.75, whereas in contrast, the Fractional Flow Reserve versus Angiography for Multivessel Evaluation (FAME) study performed coronary intervention using a cutoff FFR value of 0.80.^[1,2]^ However, the characteristics of a lesion with borderline FFR are not known. Accordingly, we evaluated the morphologic characteristics of borderline FFR using intravascular ultrasound (IVUS).

## 2 Methods

### 2.1 Study population

From January 2009 to December 2011, we enrolled 228 consecutive patients with intermediate coronary artery disease of the left anterior descending artery (LAD) who had both IVUS evaluation and measurement of FFR in our registry. We included patients with silent ischemia and stable or unstable angina who underwent elective coronary angiography. Patients were referred for elective coronary angiography if they complained of chest pain, or were positive for myocardial ischemia on a noninvasive study or routine follow-up angiography. To be included in the study, patients were required to have a *de novo* coronary artery lesion with a diameter stenosis of 40% to 70% at the proximal or mid portion of the LAD. Patients with myocardial infarction (MI), ejection fraction ≤40%, regional wall motion abnormalities on echocardiography, a lesion of restenosis, left main disease, or collateral feeding vessel were excluded. Percutaneous coronary intervention (PCI) was performed according to the operator's decision. The study protocol was approved by the institutional review board.

### 2.2 Quantitative coronary angiography

Coronary angiography was performed in the standard manner after intracoronary administration of nitroglycerin. Quantitative coronary angiography (QCA) analysis was performed using the Cardiovascular Angiography Analysis System II (Pie Medical, Maastricht, Netherlands). The percent diameter of stenosis, minimum luminal diameter, reference vessel diameter, and lesion length were measured and calculated. The lesion location was determined per the American Heart Association classification.^[11]^ An intermediate lesion was defined as 40% to 70% diameter stenosis by visual estimation and coronary artery occlusive disease was defined as ≥50% diameter stenosis. If significant stenosis was observed in the left circumflex or right coronary arteries in a patient with 2 or 3 vessel disease, we treated the lesion before conducting IVUS and FFR measurements of the LAD.

### 2.3 IVUS analyses

IVUS was performed using the Galaxy 2^TM^ IVUS System (Boston Scientific Corporation, Natick, MA) after intracoronary administration of nitroglycerin. A 40-MHz coronary imaging IVUS catheter (Atlantis SR Pro, Natick, MA) was advanced as far distally as possible in the target vessel, followed by automatic pullback at 0.5 mm/s. Off-line IVUS analyses, using software (EchoPlaque 3.0, Indec Systems, Santa Clara, CA), were performed by an independent physician blinded to the FFR value per the American College of Cardiology clinical expert consensus document on standards for acquisition, measurement, and reporting of intravascular ultrasound studies.^[12]^ Quantitative analyses included vessel, lumen, and total atheroma volumes. To standardize the vessel size, we calculated percent atheroma volume (PAV), defined as total atheroma volume divided by vessel volume × 100.

### 2.4 Measurement of FFR

FFR was measured by a 0.014-inch pressure wire ((PressureWire, Radi Medical System, Abbott Medical, St. Paul, MN) which was advanced distally to the stenosis after intracoronary administration of nitroglycerin. Measurement of FFR was performed in the standard manner.^[13]^ FFR was calculated by dividing the mean distal coronary pressure (Pd) by the mean proximal coronary pressure (Pa) during maximal hyperemia. Maximal hyperemia was induced with intracoronary continuous adenosine infusion using a microcatheter.^[14]^ We divided the patients into 3 groups by FFR value: ischemic (FFR <0.75), borderline (0.75 ≤ FFR ≤ 0.80), and nonischemic (FFR >0.80).

### 2.5 Statistical analysis

The hypothesis of this study was that the IVUS characteristics in patients with borderline FFR might be similar to FFR <0.75, however, different with patients with FFR >0.80. We did not perform power calculation because of evaluating multiple IVUS parameters. Continuous variables were presented as means ± standard deviations, and described in the following order: ischemic, borderline, and non-ischemic group. Categorical variables were presented as frequency (percentage). Continuous variables were compared using the analysis of variance test, and categorical variables were compared using the *χ*^2^ test or Fisher exact test. To assess intra- and interobserver reproducibility, we performed reliability analysis in 30 lesions. The intraclass correlation coefficients for intraobserver and interobserver reproducibility of minimal lumen area (MLA) were 0.98 and 0.96, respectively. Analysis of the clinical follow-up data was performed using a Kaplan-Meier analysis and compared with the log-rank test. Major adverse cardiac events (MACE) were defined as all-cause death, MI, and target vessel revascularization. All statistical analyses were performed using SPSS software (version 20.0, SPSS Inc, Chicago, IL). A *P* value of <.05 was considered statistically significant.

## 3 Results

Baseline clinical characteristics are shown in Table 1. We enrolled 228 patients: 27 (11.8%) patients with silent ischemia, 96 (42.1%) patients with stable angina, and 105 (46.1%) patients with unstable angina. Mean age was 61 ± 10 years, 70 (30.7%) patients had diabetes, and 134 (58.8%) patients had hypertension. A total of 106 patients had a proximal LAD lesion (46.5%) and 122 patients (53.5%) had a mid-LAD lesion. Baseline QCA and IVUS analyses are shown in Table 2. The mean reference vessel diameter was 3.34 ± 0.27 mm, the mean MLA was 3.0 ± 1.1 mm^2^, and the mean lesion length on IVUS was 20.6 ± 7.9 mm.

**Table 1:**
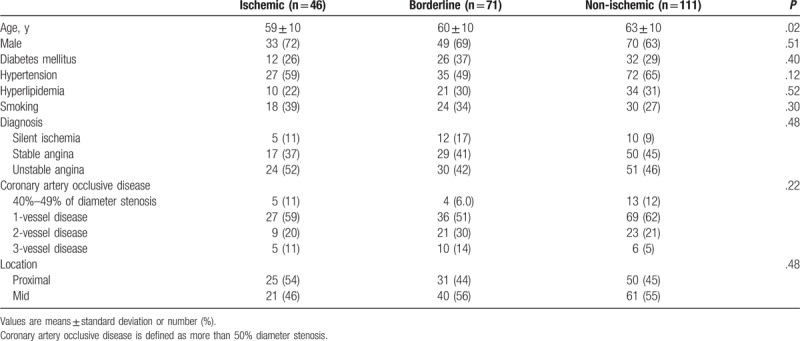
Baseline clinical and angiographic characteristics (n = 228).

**Table 2:**
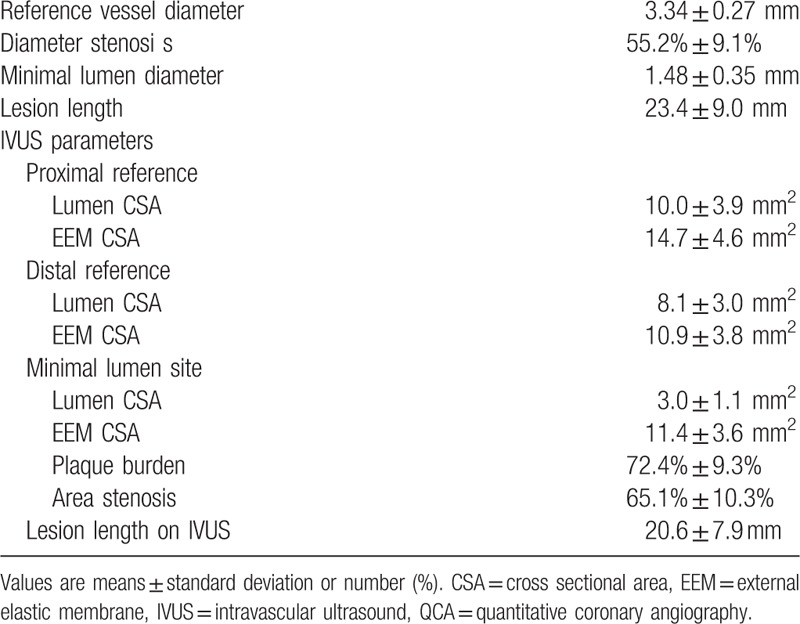
Baseline QCA and IVUS results (n = 228).

### 3.1 Comparison of QCA and IVUS analysis

There was no significant difference in mean reference vessel diameters among the 3 groups (3.3 ± 0.3 vs. 3.2 ± 0.3 vs. 3.3 ± 0.3 mm, *P* = .33). However, the diameter of stenosis was less severe (61.1 ± 8.4 vs. 57.0 ± 8.2 vs. 51.8 ± 7.4%, *P* < .001) and the MLA was larger (1.3 ± 0.3 vs. 1.4 ± 0.3 vs. 1.6 ± 0.3 mm, *P* < .001) in the nonischemic group (Table 3).

**Table 3:**
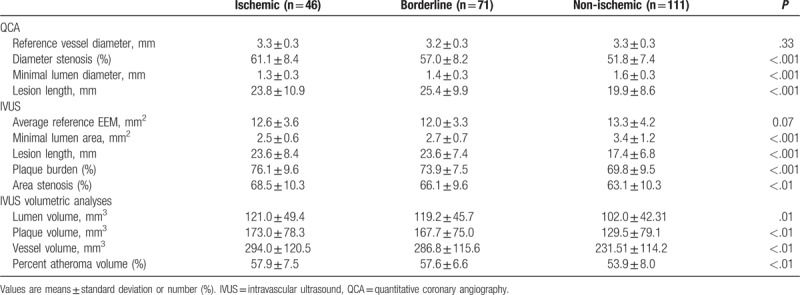
Comparison of QCA and IVUS results.

**Figure 1:**
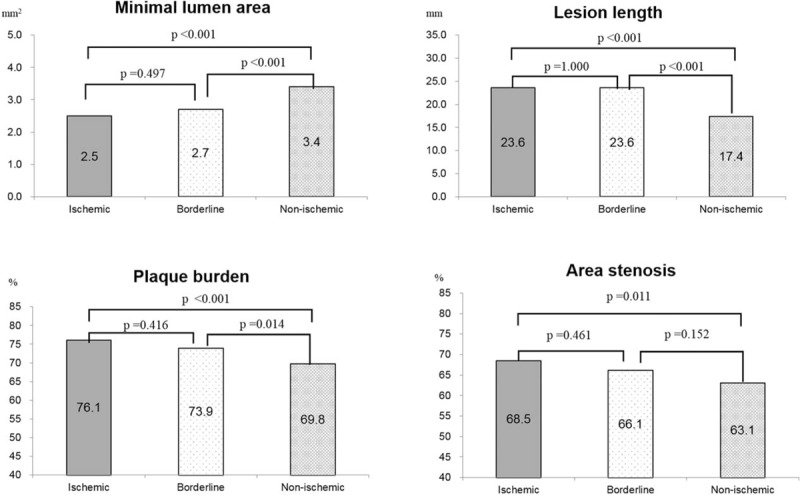
Comparison of intravascular ultrasound parameters. The minimum lumen area is smaller, and lesion length is longer, in the ischemic and borderline groups than in the non-ischemic group.

**Figure 2:**
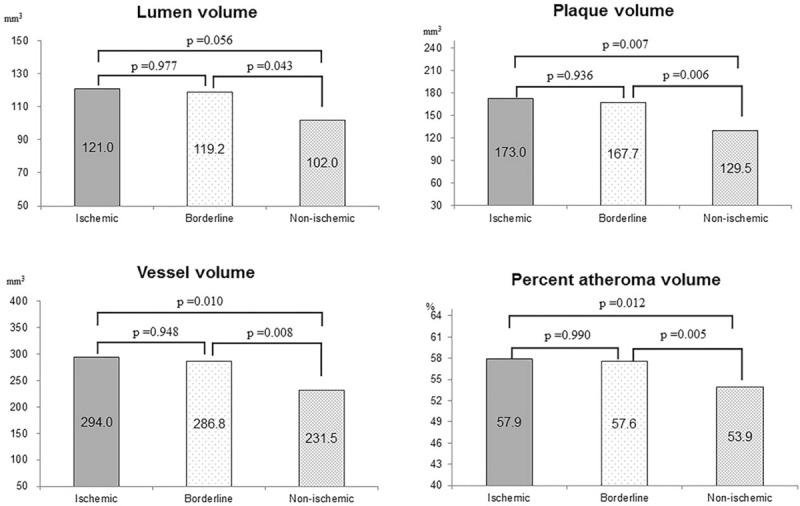
Comparison of intravascular ultrasound volumetric analysis. Plaque volume was larger and percent atheroma volume was more severe in the ischemic and borderline groups than in the non-ischemic group. There were no significant differences between the ischemic and borderline groups, but significant differences were observed between the non-ischemic and the other 2 groups.

IVUS analyses showed that the MLA was smaller (2.5 ± 0.6 vs. 2.7 ± 0.7 vs. 3.4 ± 1.2 mm^2^, *P* < .001), IVUS lesion length was longer (23.6 ± 8.4 vs. 23.6 ± 7.4 vs. 17.4 ± 6.8 mm, *P* < .001), and plaque burden (76.1 ± 9.6 vs. 73.9 ± 7.5 vs. 69.8 ± 9.5%, *P* < .001) and area of stenosis (68.5 ± 10.3 vs. 66.1 ± 9.6 vs. 63.1 ± 10.3%, *P* < .01) were more severe in the ischemic and borderline groups than in the non-ischemic group. IVUS volumetric analysis showed that the plaque volume was larger (173.0 ± 78.3 vs. 167.7 ± 75.0 vs. 129.5 ± 79.1 mm^3^, *P* < .01) and PAV was more severe (57.9 ± 7.5 vs. 57.6 ± 6.6 vs. 53.9 ± 8.0%, *P* < .01) in the ischemic and borderline groups than in the nonischemic group, respectively (Table 3). In a post-hoc analyses, there were no significant differences between the ischemic and borderline groups for all IVUS parameters, including volumetric parameters, but statistically significant differences were observed between the nonischemic and the ischemic and borderline groups (Figs. 1 and 2).

### 3.2 Clinical follow-up analysis

**Figure 3:**
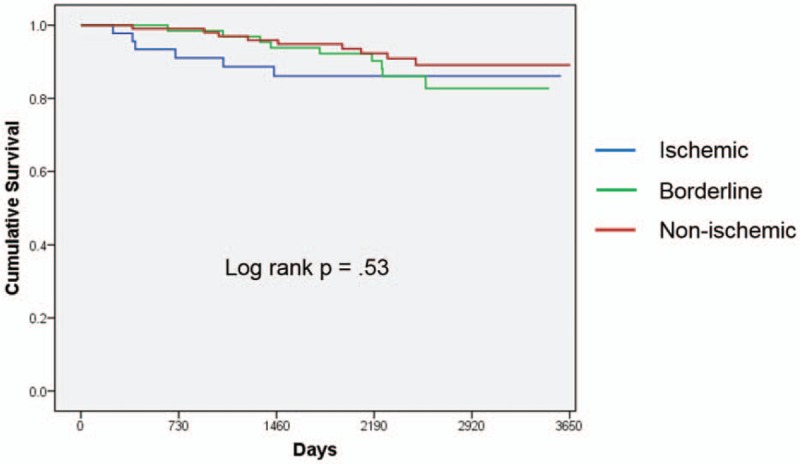
Kaplan-Meier analysis demonstrated that major adverse cardiac event was not different among 3 groups.

Mean follow-up duration was 2166 ± 838 days. The rates of MACE were 13% in the ischemic group, 13% for borderline, and 8% for the non-ischemic group up to 10 year follow-up. In the Kaplan-Meier analysis, there were no significant differences in the MACE rate among the 3 groups (*P* = .53) (Fig. 3). The rate of PCI was more frequent in the ischemic and borderline groups than in the nonischemic group (96 vs. 88 vs. 32%, *P* < .001).

## 4 Discussion

The main findings of this study are: in intermediate coronary lesions, the morphologic characteristics of borderline FFR are similar to ischemic FFR, however, different with nonischemic lesions with FFR ≥0.80.

Although the evaluation of myocardial ischemia is important, previous studies showed that noninvasive functional evaluation before PCI has been performed in less than half of patients with stable angina.^[15]^ FFR is an invasive method to evaluate the functional severity of stenosis, defined as the ratio of the pressure measured distal to the stenosis to the aorta during hyperemia, and can be easily measured in a catheterization room. FFR has been validated with several noninvasive functional studies. It has been shown that the nonischemic threshold value of >0.80 indicates an absence of inducible myocardial ischemia in the majority of patients (sensitivity 90%), whereas FFR <0.75 is associated with inducible ischemia (specificity 100%).^[4–7,16]^ However, it has been controversial as to whether borderline FFR values between 0.75 and 0.80 require revascularization. Traditionally, FFR <0.75 was considered a cutoff value for myocardial ischemia, although some investigators have reported that increasing the cutoff value to 0.80 for revascularization improved sensitivity.^[17,18]^

Although FFR-guided PCI has improved clinical outcomes in several studies, different FFR cutoff values were used. The DEFER study using FFR <0.75 showed deferral of PCI in intermediate coronary stenosis had good clinical outcomes, and performing PCI for a lesion with FFR ≥0.75 had no clinical or symptomatic benefits during a 5-year clinical follow-up.^[3]^ In contrast, the FAME 1 and 2 studies demonstrated that an FFR-guided PCI using an FFR cutoff value of 0.80 resulted in favorable clinical outcomes.^[1,2]^ Other registry data have shown that a compliance group (unrevascularized patients with FFR ≥0.80 and revascularized patients with FFR ≤0.79), using an FFR cutoff value of 0.80, provided more favorable clinical outcomes than for the noncompliance group (revascularization with FFR ≥0.80 and deferred revascularization with FFR ≤0.79).^[19]^ The DEFER study performed medical treatment patients with borderline FFR, however, in the registry data and the FAME study PCI was performed.

There were several studies that compared medical treatment with PCI treatment in patients with borderline FFR. Courtis et al have reported that medical treatment of a lesion with a borderline FFR value was associated with a higher incidence of major adverse cardiac events including a composite of cardiac death, MI, and coronary revascularization (23 vs. 5%, *P* < .01) at a mean follow-up of 13 months. These differences were mainly because of a higher incidence of coronary revascularization in the medical treatment group. Also, the medical treatment group had more frequent angina symptoms than the PCI group.^[8]^ Shiono et al reported that patients with FFR 0.75 to 0.80 were at a higher risk of target vessel failure mainly because of target vessel revascularization than those with FFR >0.80 during a 3-year follow-up.^[10]^ In contrast, Lindstaedt et al have reported that coronary lesions with borderline FFR did not increas the risk for major adverse events. They followed 97 patients with borderline FFR (defer—48, PCI—49 lesions) for a mean of 24 months. The deferred group had significantly better clinical outcomes than the revascularization group (57.1% vs. 20.8%, *P* < .01).^[9]^ However, approximately 50% of the patients had MI and the rate of MACE was high (57.1% at 2 years) compared with other PCI-related studies, so it might possibly have a selection bias.

Despite the clinical outcomes of these studies, there were no data for the characteristics of lesions with borderline FFR. Our study showed that IVUS parameters—MLA, lesion length, plaque burden, and volumetric parameters including PAV and plaque volume—of borderline FFR were similar to those of nonischemic FFR, but significantly different from those of ischemic FFR (<0.75). It is well known that IVUS-measured parameters are related to FFR. IVUS-measured MLA, lesion location, and lesion length are related to the FFR value.^[20,21]^ For the volumetric analysis, plaque volume and PAV of the target lesion had a negative correlation with FFR, and a higher plaque volume and PAV were related with functionally significant FFR.^[21]^ Previous IVUS volumetric studies have shown that PAV was related to clinical outcomes.^[22,23]^ Nicholls et al have reported that greater baseline PAVs were observed in patients who experienced MI (42.2 ± 9.6 vs. 38.6 ± 9.1%, *P* < .01), coronary revascularization (41.2 ± 9.3 vs. 38.1 ± 9.0%, *P* < .001), or MACE (41.3 ± 9.2 vs. 38.0 ± 9.0%, *P* < .001).^[22]^ The Study of Coronary Atheroma by Intravascular Ultrasound: Effect of Rosuvastatin vs. Atorvastatin (SATURN) study showed that patients with the highest quartile of baseline PAV had a significantly higher 2-year cumulative MACE rate than those in the lower PAV quartiles (12% vs. 5.1%, *P* < .01).^[23]^ As the morphologic characteristics of borderline FFR are similar to ischemic FFR and IVUS-measured parameters are related with clinical outcomes, a cutoff point FFR of 0.80 seems to be appropriate criteria for coronary revascularization in intermediate lesions of the LAD.

This study had several limitations. First, this was a retrospective study. We did not randomize the treatment methods according to the FFR value. Therefore, the clinical outcomes of the 3 groups could not be evaluated accurately. Moreover, the number of lesions was too small to robustly assess clinical outcomes. Second, as we included only LAD lesions, our conclusion may not be applicable to all coronary vessels. However, this was also a strength of our study in terms of only evaluating homogenous lesions to precisely determine the relationship between IVUS and FFR. Third, although many cardiologists accept that an FFR ≤0.80 indicates a critical ischemic state, the data for clinical outcomes in patients with borderline FFR remain controversial. Further study of IVUS parameters in lesions with borderline FFR will be needed. Finally, among the IVUS parameters, we did not assess plaque vulnerability, such as soft plaque, plaque with a large lipid core, or plaque rupture. As well as plaque volume, plaque vulnerability is an important predictor for acute coronary syndrome.

## 5 Conclusion

There were no differences in IVUS characteristics between borderline and functionally significant FFR, but the stenosis and amount of atheromatous plaque were more severe in these 2 groups than in patients with nonischemic lesions with FFR ≥0.80 in intermediate coronary lesions.

## Author contributions

**Conceptualization:** Hyoung-Mo Yang, Hong-Seok Lim.

**Data curation:** Hong-Seok Lim, Kyoung-Woo Seo, Byoung-Joo Choi, So-Yeon Choi, Myeong-Ho Yoon, Gyo-Seung Hwang, Seung-Jea Tahk.

**Formal analysis:** Hyoung-Mo Yang, Hong-Seok Lim, Kyoung-Woo Seo, Byoung-Joo Choi, So-Yeon Choi, Myeong-Ho Yoon, Gyo-Seung Hwang, Seung-Jea Tahk.

**Funding acquisition:** Seung-Jea Tahk.

**Investigation:** Hong-Seok Lim, Kyoung-Woo Seo, Byoung-Joo Choi, So-Yeon Choi, Myeong-Ho Yoon, Gyo-Seung Hwang, Seung-Jea Tahk.

**Methodology:** Hong-Seok Lim, Kyoung-Woo Seo, So-Yeon Choi, Myeong-Ho Yoon, Gyo-Seung Hwang, Seung-Jea Tahk.

**Writing – original draft:** Hyoung-Mo Yang.

**Writing – review & editing:** Hyoung-Mo Yang, Hong-Seok Lim, Kyoung-Woo Seo, So-Yeon Choi, Myeong-Ho Yoon, Seung-Jea Tahk.
